# Medical Students in 1849

**Published:** 1849-12

**Authors:** 


*Medical Students in* 1849.—It is a singular fact, and not a very encouraging one for the already over-crowded profession in France, that, in spite of the perturbed condition of that country, the number of inscriptions at the Faculty of Paris, for the year 1849, very considerably exceeds that of any of the prior five years, being 272, while it has risen from 216 to 251 in those years. The total number of pupils now in course of education in Paris, amounts to 950, while in 1845 it was but 800. The other faculties do not, however, participate in the increase; as the number of students this year, at Strasburg. is but 109, being just the same as in 1845; and at Montpellier, 174, *vice* 173. The *Ecoles preparatories* at the different large towns, vary much in respect to the number of pupils educating at them. Thus, at Lyons there are 81, Toulouse 76, Marseilles 48, Rouen 45, Bordeaux 38, at other places from 24 to 26, while Rheims only musters 15—rather a small number for a staff of *eight* professors. The total number of the pupils at these schools amounts to 792, and was 751 in 1845. —*Rev. Med. Chir.* t. v. p. 316.

